# Eosinophile Ösophagitis – Eine unterschätzte Nahrungsmittelallergie?

**DOI:** 10.1007/s00105-025-05513-0

**Published:** 2025-05-26

**Authors:** A. Hörning, A. Madisch

**Affiliations:** 1 Pädiatrische Gastroenterologie, Hepatologie und Endoskopie, Klinik für Kinder- und Jugendmedizin, Universitätsklinikum Erlangen, Friedrich-Alexander-Universität Erlangen-Nürnberg, Loschgestr. 15, 91054 Erlangen, Deutschland; 2 Zentrum Innere Medizin, Diakovere Friederikenstift, Hannover, Deutschland

**Keywords:** Eliminationsdiät, Nahrungsmittelallergene, Eosinophile Infiltration, Speiseröhrenfunktionsstörung, Lebensqualität, Elimination diet, Food allergens, Eosinophilic infiltration, Esophageal dysfunction, Quality of life

## Abstract

Die eosinophile Ösophagitis (EoE) gehört zu den chronischen Erkrankungen mit T2-inflammatorischer Pathophysiologie und kann im übertragenen Sinne als atopische Dermatitis der Speiseröhre bezeichnet werden. Sie ist eine durch Nahrungsmittelallergene immunvermittelte Erkrankung, die histologisch durch eine eosinophile Infiltration definiert und klinisch durch Symptome der Speiseröhrenfunktionsstörung gekennzeichnet ist. Neben der Reduktion der derzeit noch erheblichen diagnostischen Latenz steht die Priorisierung bestehender und neuer Therapieoptionen im Fokus mit dem Ziel, eine effektive Patientenversorgung mit verbesserter Lebensqualität langfristig zu gewährleisten.

## Abstract (en)

Eosinophilic oesophagitis (EoE) is a chronic T2-inflammatory disease and can be figuratively referred to as atopic dermatitis of the oesophagus. It is an immune-mediated disease caused by food allergens and is histologically defined by eosinophilic infiltration and clinically characterised by symptoms of oesophageal dysfunction. In addition to reducing the currently still considerable diagnostic latency, the focus is on prioritising existing and new treatment options with the aim of ensuring effective patient care with improved quality of life in the long term.

Seit der Erstbeschreibung in den frühen 1990er-Jahren hat sich die eosinophile Ösophagitis (EoE) aufgrund steigender Prävalenz von einer kasuistisch beschriebenen Rarität nach der gastroösophagealen Refluxerkrankung zu einer der häufigsten entzündlichen Erkrankungen der Speiseröhre entwickelt [1, 2]. In Europa wird die Inzidenz der EoE bei Erwachsenen auf 4,1 pro 100.000 Einwohner und bei Kindern auf 3,5 pro 100.000 geschätzt, mit einer Prävalenz von 32,7 pro 100.000 bei Erwachsenen und 18,1 pro 100.000 bei Kindern [3]. Die EoE kann in jedem Lebensalter auftreten, das männliche Geschlecht ist 2‑ bis 3‑mal häufiger betroffen. Auf dem Boden einer epithelialen Barrierestörung sind Nahrungsmittelproteine aus Kuhmilch, Weizen, Hühnerei, Soja, Meeresfrüchte und Nüsse als Auslöser der EoE zu sehen. Als begünstigende Risikofaktoren werden schädigende Umweltfaktoren wie Mikroplastik, Ozon, Detergenzien oder Frühgeburtlichkeit und Sectio caesarea diskutiert. Es gibt keine standardisierten Labortests zur Identifikation der auslösenden Allergene. Der enge Zusammenhang zwischen EoE und allergischen Komorbiditäten hat dazu geführt, dass die EoE als Spätmanifestation des allergischen Marsches diskutiert wird [4].

Unbehandelt nimmt die EoE in der Regel den Verlauf einer chronisch persistierenden Entzündung, die zu einem bindegewebigen Umbau des Ösophagus mit Strikturen und Funktionsstörungen führen kann [5]. Eine frühe Diagnose und Einleitung einer effektiven Therapie ist daher essenziell, um schwerwiegende Komplikationen zu verhindern [6].

Die EoE ist pathophysiologisch abzugrenzen von der gastroösophagealen Refluxkrankheit (GERD), beide können koexistieren, aber sich auch bidirektional beeinflussen [7]. So kann die GERD über eine Verletzung der mukosalen Integrität zu einer gesteigerten transepithelialen Allergenpermeabilität mit nachfolgender allergenvermittelter Immunaktivierung an der Pathogenese der EoE beteiligt sein [8].

Es bestehen eine familiäre Häufung und eine genetische Prädisposition für die EoE

Die EoE wird durch TH2-Helferzellen vermittelt und ist Ig(Immunglobulin)E unabhängig [9]. Die EoE-Pathogenese ist als multifaktorieller Prozess zu verstehen und in Abb. 1 zusammengefasst. Die EoE, die atopische Dermatitis und das allergische Asthma bronchiale teilen ein ähnliches Muster krankheitsspezifischer Transkripte, was eine gemeinsame molekulare Ätiologie hervorhebt [10].

**Abb. 1:**
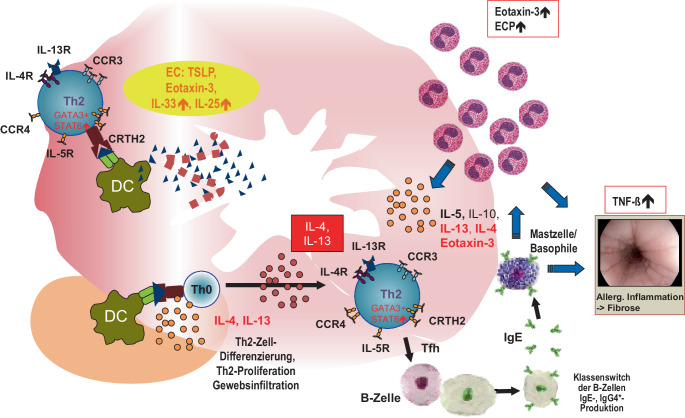
Eine Barrierestörung der Ösophagusschleimhaut führt zur transepithelialen Deposition von Nahrungsmittelallergen. Die Epithelzellen der Speiseröhre schütten Alarmine (u. a. TSLP [Thymus-Stroma-Lymphopoietin], IL[Interleukin]-33, IL-25) sowie Chemokine (z. B. Eotaxin-3) aus, das die allergische Entzündungsreaktion triggert. Ortsständige dendritische Zellen werden aktiviert, steigern die Expression der Zytokine IL‑5, IL-13 und IL-15 und prozessieren und präsentieren das Nahrungsmittelallergen den durch Chemotaxis rekrutierten TH2-Zellen, die gemeinsam mit den Epithelzellen die ösophageale Eosinophilenimmigration vermitteln. Eine weitere Differenzierung und Aktivierung von TH2-Zellen findet in lokoregionalen Lymphknoten statt, welches die allergisch-entzündliche Reaktion verstärkt. Letztendlich werden zunehmend Eosinophile in der ösophagealen Schleimhaut rekrutiert, es bildet sich bei Chronifizierung dann ein Mischinfiltrat aus zahlreichen Immunzellen (wie z. B. Basophile, CD8^+^-T-Zellen und Mastzellen). Letztlich werden auch im Rahmen des allergischen TH2-Entzündungsweges B‑Zellen durch CXCR5^+^-TH-Zellen aktiviert, die zu Plasmablasten und Ig(Immunglobulin)E produzierenden Plasmazellen heranreifen und zum Epiphänomen der nahrungsmittelspezifischen IgE-Bildung führen. IL‑5 und IL-13 induzieren wiederum in der Ösophagusmukosa eine ausgeprägte Überexpression des potenten Eosinophilen-Chemokins Eotaxin‑3. Eosinophile setzen u. a. eosinophile Peroxidase (EPO) und das eosinophile kationische Protein (ECP) frei, die direkt weitere Mukosaschäden und eine Dysmotilität der Speiseröhre verursachen. Ferner verringert IL-13 die Barrierefunktion des Epithels, indem es die Genexpression des Hauptstrukturproteins Filaggrin reduziert, das für die Erhaltung der epithelialen Integrität entscheidend ist. Wie bei der atopischen Dermatitis tragen TH2-Zytokine dazu bei, die Epithelbarriere weiter zu schädigen, was wiederum zur Etablierung einer Sensibilisierung gegenüber Nahrungsmittelallergenen führt, die dann letztlich bei der Mehrzahl der Patienten den chronischen Auslöser der Erkrankung darstellen. (Mit freundl. Genehmigung, © A. Hörning)

Es bestehen eine familiäre Häufung und eine genetische Prädisposition für die EoE [11, 12]. Männliche Verwandte 1. Grades haben ein bis zu 64faches erhöhtes Risiko, eine EoE zu entwickeln [11]. Ein- und zweieiige Zwillinge waren in 41 % bzw. 22 % der Fälle an einer EoE erkrankt. Es wurden genetische Polymorphismen für die EoE identifiziert, die Überlappungen mit assoziierten Genloci anderer atopischer Erkrankungen aufweisen, z. B. TSLP (Thymus-Stroma-Lymphopoietin), CCL26 (Eotaxin-3) Filaggrin (FLG), Desmoglein (DSG1) und CAPN14 [10, 13]. Kinder mit atopischen Vorerkrankungen haben ein erhöhtes Risiko, eine EoE zu entwickeln, in diesen Fällen scheint die EoE als Erkrankung des allergischen Formenkreises sich zum späten Zeitpunkt des „atopic march“ zu manifestieren [4]. Die Prävalenz atopischer Begleiterkrankungen ist bei EoE-Patienten häufiger als in der Normalbevölkerung und liegt bei Kindern zwischen 42 und 96 % [14]. Dazu gehören das Asthma bronchiale (ca. 40 % der Fälle), die Neurodermitis (ca. 30 % der Fälle), die Pollinosis (ca. 60 % der Fälle) [5]. Letztlich weist zudem eine bedeutende Anzahl von Kindern mit operierter Ösophagusatresie (ca. 15 % der Fälle) eine EoE auf [5, 15].

Der chronische Verlauf, das diagnostische Dilemma mit der Notwendigkeit engmaschiger klinischer und endoskopisch-histologischer Verlaufskontrollen wirken sich negativ auf die gesundheitsbezogene Lebensqualität bei Kindern und Erwachsenen aus [16–18]. Bolus- und Erstickungsängste können den Lebensalltag belasten, Angstzustände und Depressionen sind die Folge [19, 20].

Die klinische Präsentation der EoE ist bei Kindern und Jugendlichen/Erwachsenen sehr unterschiedlich. Während die häufigsten Symptome bei Jugendlichen und Erwachsenen durch Dysphagie (70–80 %) und Bolusobstruktion (33–54 %) gekennzeichnet sind [21, 22], sind die Symptome bei Kleinkindern und Kindern oftmals uneinheitlich. Bei Säuglingen und Kleinkindern finden sich eher unspezifische Symptome wie Reflux-ähnliche Beschwerden mit Erbrechen (27 %), Übelkeit (27 %) oder schlichtweg Nahrungsverweigerung (14 %) und ggf. eine Gedeihstörung. Dysphagie (28 %) und Bolusobstruktionen (7 %) treten insbesondere bei Jugendlichen auf ([23, 24]; Tab. 1).

**Tab. 1:** Klinische Symptome der eosinophilen Ösophagitis (EoE) im Kindesalter [24]

Häufigste Symptome	Auftreten (Lebensalter in Jahren), *n* = 103
Fütterungsstörung 14 %	Median 2 Jahre
Erbrechen 27 %	Median 8 Jahre
(Ober‑)Bauchschmerz/retrosternaler Schmerz 27 %	Median 12 Jahre
Dysphagie 28 %	Median 13 Jahre
Bolusverlegung der Speiseröhre 7 %	Median 17 Jahre

## Merksatz.

**Abb. 2:**
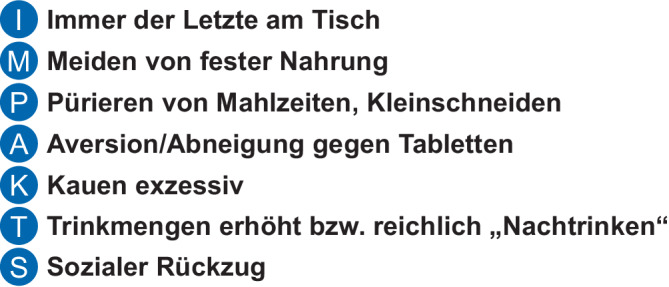
„Disease awareness“: Um Beschwerden beim Essen zu vermeiden, entwickeln viele Patienten adaptive Strategien, wie unter dem Akronym IMPAKTS zusammengefasst. Daher sollten auch gezielte Fragen nach dem Essverhalten der Patienten Bestandteil der Anamnese sein, damit das Ausmaß des Beschwerdebildes genauer erfasst werden kann. (Mit freundl. Genehmigung, © A. Madisch)

Gezieltes Fragen nach dem Essverhalten der Patienten sollte Bestandteil der Anamnese sein, denn angepasstes Verhalten bzw. Gewöhnung über die Zeit kann Symptome maskieren und resultiert in einer verzögerten EoE-Diagnose (Abb. 2).

**Abb. 3:**
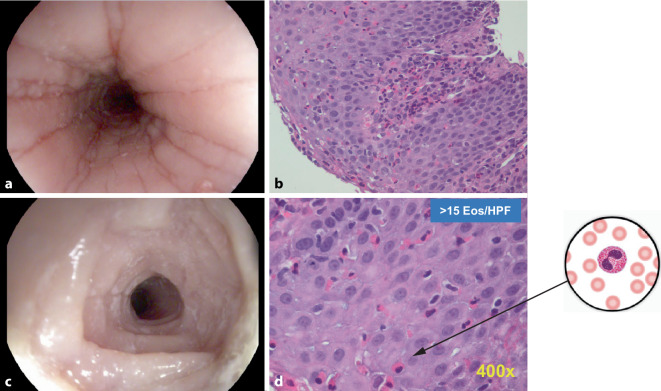
Endoskopischer Untersuchungsbefund und histologisches Bild der eosinophilen Ösophagitis (EoE) bei einem 15-jährigen Jungen. **a** Längsfurchen, Schleimhautödem mit fehlender Gefäßzeichnung und Präzipitaten (am rechten unteren Bildrand), **b** Übersicht ösophagealen Biopsiegewebes in Hämatoxilin-Eosin-Färbung mit Basalzellhyperplasie und Nachweis der Infiltration eosinophiler Granulozyten, **c** Ringfurchung mit langstreckiger Lumeneinengung im distalen Bereich des Ösophagus, **d** 400-fache Vergrößerung erlaubt die Quantifizierung mit Überschreiten des Grenzwertes > 15 Eos/HPF. *Eos* Eosinophile, *HPF* hochauflösendes Gesichtsfeld. (**a**, **c** mit freundl. Genehmigung, © A. Madisch, A. Hörning; **b**, **d** mit freundl. Genehmigung, © PD Dr. P. Rümmele, Institut für Pathologie, UK Erlangen)

Die Diagnose der EoE wird histologisch gestellt, eine Eosinophilenzahl von > 15 pro hochauflösendem Gesichtsfeld (HPF, Standardgröße 0,3 mm^2^) gilt als diagnostischer Schwellenwert für eine EoE (Abb. 3c, d). Endoskopisch sichtbare strukturelle Veränderungen der Speiseröhre, die mit der EOE einhergehen, sind weißliche Exsudate (entsprechen eosinophilen Mikroabszessen), Längsfurchen und ein Schleimhautödem (Abb. 3a). Während diese als Zeichen der akuten Entzündung zu werten sind, spiegeln eine fixierte Ringbildung (sog. Trachealisierung oder Felinisierung der Speiseröhre), ein kleinkalibriges Ösophaguslumen und umschriebene Strikturen ein chronisches Fibrosestadium wider (Abb. 3c, 2b; [25, 26]). Diese makroskopischen Befunde werden in der Erwachsenenmedizin, aber auch in der Kinder- und Jugendmedizin anhand des gültigen Bewertungssystems nach Hirano (EREFS[Exsudate, Ringe, Ödeme, Furchen, Strikturen]-Klassifikation [27]) berücksichtigt, sie können allein oder in Kombination auftreten.

Für die Diagnosestellung der EoE und deren Verlaufsbeurteilung ist die Entnahme von Stufenbiopsien des Ösophagus (oberes, mittleres und unteres Drittel) zwingend erforderlich. Diese müssen in jedem Fall durchgeführt werden, weil auch unauffällige Endoskopiebefunde bei Kindern und Erwachsenen mit EoE in 32 % der Fälle beobachtet werden [28, 29]. Zudem weist die eosinophile Entzündung des Ösophagus ein unregelmäßig verteiltes Befallsmuster auf („patchy disease“) [30, 31]. Daher wird die diagnostische Sensitivität durch die Entnahme mehrerer Biopsien aus mindestens 2 oder mehr Abschnitten des Ösophagus gesteigert [32].

## Therapieziel

Die aktive EoE geht mit einer chronischen, eosinophilen prädominanten Inflammation des Ösophagus, chronisch rezidivierenden ösophagealen Symptomen und einer signifikant reduzierten Lebensqualität einher. Bleibt die Erkrankung unbehandelt besteht zudem ein hohes Risiko für ösophageale Fibrose, Strikturen und Bolusobstruktionen. Aus diesen Gründen soll bei Nachweis einer aktiven EoE eine effektive Induktionstherapie begonnen werden mit dem Ziel, eine klinisch-histologische Remission zu erreichen [6].

Bei Nachweis einer aktiven EoE soll eine effektive Induktionstherapie begonnen werden

Für die Überprüfung des Vorliegens einer histologischen Remission eignet sich momentan nur die Endoskopie mit Biopsie, denn Symptome sowie endoskopische Befunde korrelieren oftmals selbst bei Erwachsenen nur mangelhaft mit der entzündlich-histologischen Aktivität [33, 34]. Bislang sind keine verlässlichen non-invasiven Biomarker identifiziert worden. Die Überprüfung der Induktionstherapie erfolgt nach 8 bis 12 Wochen [6]. Aufgrund des chronisch progressiven Charakters sollten EoE-Patienten nach erfolgreicher Induktionstherapie eine dauerhafte remissionserhaltende Therapie erhalten [6]. Diese kann prinzipiell in der Fortsetzung der medikamentösen Therapie, wenn möglich in halbierter Dosis, oder in einer Langzeitelimination von identifizierten Nahrungsmittelallergenen geschehen [6]. Eine Reprovokation mit anschließender endoskopisch-histologischer Evaluation (nach 8 bis 12 Wochen) ist im Kleinkindesalter nach 1 Jahr der Elimination sinnvoll, um Toleranzentwicklung zu prüfen.

## Therapieprinzip und therapeutisches Vorgehen

Gemäß aktuellen europäischen Empfehlungen sowie der neuen deutschen S2k-Leitlinie [35] sollen Patienten mit gesicherter EoE eine topische Steroidtherapie und/oder eine Eliminationsdiät angeboten werden. Ein an die aktuelle Evidenzlage angepasster Therapiealgorithmus für das Kindes- und Erwachsenenalter ist vor Kurzem veröffentlicht worden [6].

Die *Therapie mittels Ernährung *reicht von einer Elimination der häufigsten Nahrungsmittelallergene als 1‑, 2‑, 4‑ oder 6‑Food-Eliminationsdiät bis zur exklusiven aminosäurebasierten Ernährung. Komplizierend für die diagnostische Identifikation des auslösenden Allergens ist, dass gerade im jungen Kleinkindes- und Schulkindesalter der Auslöser der EoE in Nordeuropa zumeist durch 1 bis 3 Nahrungsmittelallergene (Kuhmilch, Weizen, Hühnerei) bedingt ist und sowohl das einzelne, aber auch deren Kombination entsprechend schwer zu spezifizieren ist [36–38].

Die empirische 6‑Food-Elminationsdiät (6FED) (Elimination von Kuhmilchprotein, Weizen/Gluten, Ei, Soja, Nüsse, Fisch und Meeresfrüchte) ist gut untersucht und führt zuverlässig bei rund zwei Drittel der Patienten (Kinder und Erwachsene) zu histologischer Remission [6, 39]. Der Vorteil bei dieser sehr restriktiven Diät liegt in der Möglichkeit der spezifischen Identifikation der einzelnen auslösenden Nahrungsmittelallergene, denn die anschließende Elimination führt zur Abheilung der Ösophagitis. Die diätetischen Einschränkungen sind allerdings erheblich, beeinträchtigen die Lebensqualität und stellen die Betroffenen vor große Herausforderungen.

Zudem wird zwischen einer Top-down-Strategie (d. h. zunächst 6FED, dann Nachweis histologischer Remission, gefolgt von schrittweiser Wiedereinführung, jeweils mit Endoskopie) und einer Step-up-Therapie (zumeist mit Elimination von Kuhmilch allein oder Kuhmilch und Weizen als 2FED und Eskalation bei weiterbestehendem histologischem Bild) unterschieden. Bei Wiedereinführung führen Kuhmilch in 85 %, Hühnerei in 25 %, Weizen in 33 % und Soja in 19 % zum Wiederaufflammen der Entzündung [40]. Da allerdings die überwiegende Mehrzahl der Patienten mit Ansprechen auf Ernährungstherapie nur 1 oder 2 Nahrungsmitteltrigger aufweist, ist die 6FED in vielen Fällen nicht notwendig. Inzwischen wird oft eine initiale 1FED, 2FED oder 4FED empfohlen, hierbei werden zumeist Kuhmilch und/oder Weizen und Hühnerei sowie Soja eliminiert. Dann wird schrittweise wiedereingeführt und endoskopisch histologisch der Therapieerfolg geprüft. Die Effektivität für das Erreichen der klinischen und histologischen Remission liegt mit Berücksichtigung der wenigen RCT(„randomized controlled trial“)-Daten bei der 1FED, 2FED und 4FED respektive bei 34–46 % bzw. 44 % und 41–55 % [6].

Die Elementarernährung mit aminosäurebasierten Trinknahrungen stellt die einfachste, aber auch am stärksten einschränkende Ernährungstherapie dar. Sie ist aufgrund der ausgeprägten diätetischen Einschränkungen, des unzureichenden Geschmacks und der damit verbundenen reduzierten Compliance vorwiegend zur Überbrückung bzw. bei Säuglingen oder bei Patienten mit sondenabhängiger Ernährung geeignet. Sie ist mit einer histologischen Remission bei über 90 % der Patienten innerhalb von wenigen Wochen als sehr effektiv einzustufen [6].

Obwohl bei Ernährungstherapie keine unerwünschten Arzneimittelwirkungen zu erwarten sind, besteht dennoch ein Risiko zur negativen Beeinflussung des kindlichen und familiären Essverhaltens, das mit einer Ernährungsstörung assoziiert sein kann [41]. In allen Fällen sollte bereits bei initialer Diagnosestellung eine qualifizierte Ernährungsfachkraft, die auch in der Therapie von Nahrungsmittelallergien versiert ist, involviert werden, da insbesondere im Kindes- und Jugendalter eine Gedeihstörung vermieden oder ggf. behoben werden muss.

Die Wirksamkeit einer Induktionstherapie mit *topischen Steroiden* (Budesonid-Schmelztablette mit Zulassung ab dem 18. Lebensjahr) ist durch mehrere randomisierte, placebokontrollierte Studien und Metaanalysen gut belegt und führt mit einer Dosierung von 2‑mal täglich mit jeweils 1 mg in einem hohen Prozentsatz zu einer histologischen und klinischen Remission [6]. Auch in der Langzeittherapie ist ein niedrig dosiertes Budesonid im Erwachsenenalter effektiv und sicher. Hier liegen mittlerweile aktuelle und positive Langzeitstudiendaten (48 Wochen doppelblinde Phase, gefolgt von 96 Wochen offene Therapie mit Budesonid 2‑mal 0,5 mg) für die orodispersible Budesonid-Tablette vor, die bei sehr guten Remissionsraten > 80 % auch eine gleichbleibende gute Verträglichkeit zeigte ohne neue Sicherheitssignale. Sicherheitsdaten für das Kindes- und Jugendalter liegen hinsichtlich einer mehrjährigen Langzeittherapie bislang nicht vor.

Die wissenschaftliche Datenlage zur Wirksamkeit von Protonenpumpeninhibitoren (PPI) in der EoE-Therapie ist dagegen im Vergleich zu den topischen Glukokortikoiden deutlich schwächer, da placebokontrollierte Studien fehlen. Publizierte Metaanalysen von prospektiven und retrospektiven Fallsammlungen geben Remissionsraten von bis zu 50 % an [6]. Der Wirkmechanismus beim PPI scheint hierbei nicht nur an der antaziden Wirkung zu liegen, denn auf molekularer Ebene konnte gezeigt werden, dass die Expression relevanter Gene (u. a. von Eotaxin-3) im Ösophagusepithel reduziert wird [42, 43]. Die PPI-Therapie kann in der Therapie der EoE insbesondere bei Kindern erwogen werden, ist in Deutschland aber zur Behandlung der EoE nicht zugelassen.

Bei unzureichender Wirkung der vorgenannten Substanzen steht das Biologikum Dupilumab (IL[Interleukin]-4Rα/IL-13Rα1-Inhibitor) mit Arzneimittelzulassung ab dem 2. Lebensjahr zur Verfügung [6, 44]. Das Erreichen der klinischen und histologischen Remission liegt für das Dupilumab (Anti-IL-4Rα/IL-13Rα1) bei gutem Sicherheitsprofil zwischen 60 und 86 % [6].

Da es sich bei der eosinophilen Ösophagitis um eine chronische Erkrankung handelt, ist eine langfristige Remissionserhaltungstherapie mit dem Therapieprinzip, mit dem die Remission erreicht wurde, erforderlich.

### Merksatz.

Bei aktiver nicht strikturierender EoE sollte zur Remissionsinduktion (klinisch und histologisch) im Erwachsenenalter primär eine orale Budesonid-Therapie erfolgen, im Kindes- und Jugendalter je nach Alter und atopischer Komorbidität eine Eliminations- oder Elementardiät, eine PPI- oder Budesonid-Therapie. Im Falle eines Therapieversagens dieser Optionen sollte Dupilumab ab dem 2. Lebensjahr eingesetzt werden.

## Fazit für die Praxis

- Für die eosinophile Ösophagitis (EoE) stehen mittlerweile wirksame Therapieoptionen zur Verfügung. Dennoch bleiben Fragen offen, u. a. in Bezug auf den langfristigen Krankheitsverlauf, die Dauer der Remissionserhaltungstherapie und die Ermittlung nichtinvasiver Marker zur Einschätzung der Krankheitsaktivität.
- Die EoE sollte bei atopischer (Ko‑)Morbidität unbedingt anamnestisch berücksichtigt werden, Verzögerungen bei der Diagnose gilt es zu vermeiden.
- Eine angemessene Behandlung und langfristige Betreuung der betroffenen Patienten sind notwendig, um ihnen eine optimale Lebensqualität zu sichern.
